# The structure of the leukemia drug imatinib bound to human quinone reductase 2 (NQO2)

**DOI:** 10.1186/1472-6807-9-7

**Published:** 2009-02-24

**Authors:** Jonathan A Winger, Oliver Hantschel, Giulio Superti-Furga, John Kuriyan

**Affiliations:** 1 Department of Molecular and Cell Biology, California Institute for Quantitative Biosciences (QB3), Howard Hughes Medical Institute, University of California, Berkeley, CA, USA; 2 Department of Chemistry, California Institute for Quantitative Biosciences (QB3), Howard Hughes Medical Institute, University of California, Berkeley, CA, USA; 3 Center for Molecular Medicine of the Austrian Academy of Sciences, Vienna, Austria

## Abstract

**Background:**

Imatinib represents the first in a class of drugs targeted against chronic myelogenous leukemia to enter the clinic, showing excellent efficacy and specificity for Abl, Kit, and PDGFR kinases. Recent screens carried out to find off-target proteins that bind to imatinib identified the oxidoreductase NQO2, a flavoprotein that is phosphorylated in a chronic myelogenous leukemia cell line.

**Results:**

We examined the inhibition of NQO2 activity by the Abl kinase inhibitors imatinib, nilotinib, and dasatinib, and obtained IC_50 _values of 80 nM, 380 nM, and >100 μM, respectively. Using electronic absorption spectroscopy, we show that imatinib binding results in a perturbation of the protein environment around the flavin prosthetic group in NQO2. We have determined the crystal structure of the complex of imatinib with human NQO2 at 1.75 Å resolution, which reveals that imatinib binds in the enzyme active site, adjacent to the flavin isoalloxazine ring. We find that phosphorylation of NQO2 has little effect on enzyme activity and is therefore likely to regulate other aspects of NQO2 function.

**Conclusion:**

The structure of the imatinib-NQO2 complex demonstrates that imatinib inhibits NQO2 activity by competing with substrate for the active site. The overall conformation of imatinib when bound to NQO2 resembles the folded conformation observed in some kinase complexes. Interactions made by imatinib with residues at the rim of the active site provide an explanation for the binding selectivity of NQO2 for imatinib, nilotinib, and dasatinib. These interactions also provide a rationale for the lack of inhibition of the related oxidoreductase NQO1 by these compounds. Taken together, these studies provide insight into the mechanism of NQO2 inhibition by imatinib, with potential implications for drug design and treatment of chronic myelogenous leukemia in patients.

## Background

**Figure 1:**
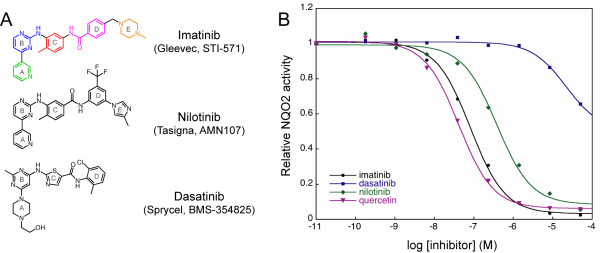
**Inhibition of NQO2 by Abl kinase inhibitors**. A) Chemical structures of the Abl kinase inhibitors imatinib, nilotinib, and dasatinib. Imatinib consists of a pyridine ring (A, *green*), an aminopyrimidine ring (B, *blue*), a methylbenzene ring (C, *red*), a benzamide ring (D, *magenta*), and a N-methylpiperazine ring (E, *orange*). The structurally analogous rings of nilotinib and dasatinib are similarly labeled. B) NQO2 inhibition assays for kinase inhibitors imatinib (*black circles*), nilotinib (*blue squares*), dasatinib (*green diamonds*), and the flavonoid NQO2 inhibitor quercetin (*magenta triangles*). The data were fit to the concentration-response equation $\text{activity}=\min+\frac{\max-\min}{1+10^{\left(x-\log{\text{IC}}_{50}\right)}}$, where x is the log of the inhibitor concentration, to yield IC_50 _values of 42 nM, 82 nM, and 381 nM for quercetin, imatinib, and nilotinib, respectively. Dasatinib was a very poor inhibitor, with an IC_50 _value > 100 μM.

Chronic myelogenous leukemia (CML) is caused by expression of a single oncoprotein resulting from the fusion of the BCR and ABL genes [1]. The Abl protein is a ubiquitously-expressed tyrosine kinase involved in multiple signaling pathways, and the fusion of the Bcr protein to the N-terminus of Abl in hematopoietic stem cells results in an oncoprotein with unregulated tyrosine kinase activity [2]. This causes cell proliferation, ultimately leading to leukemic transformation [3]. Imatinib (Gleevec, STI-571) is a 2-phenylaminopyrimidine compound (Figure 1A) that represents the first in a class of targeted anticancer drugs developed to treat CML through inhibition of Bcr-Abl [4]. Due to the large number of kinases in the human genome and the structural conservation of the kinase catalytic domain, targeting specific kinases has been particularly difficult. Nevertheless, imatinib is remarkably specific, and is effective against a very limited set of tyrosine kinases, including Kit, PDGFR and DDR in addition to Abl [5].

A series of biochemical and structural studies have elucidated the mechanisms responsible for the inhibition of Abl by imatinib. Protein kinases generally adopt similar active conformations, but can differ significantly in their inactive conformations; imatinib inhibits Abl specifically by binding to an inactive kinase domain conformation that is characteristic of Abl [6-8]. The Kit kinase domain also adopts an inactive conformation when bound to imatinib, and this conformation resembles that of Abl bound to imatinib [9,10]. Kit and PDGFR are now therapeutic targets of imatinib for tumor types in which they are in a deregulated state [11,12].

Imatinib displays excellent efficacy and minimal side effects in clinical studies with CML patients [13,14], and now represents the frontline therapy for CML [15]. However, patients in advanced stages of the disease develop resistance to imatinib treatment, due to the acquisition of mutations in the Abl kinase domain that render the protein insensitive to this inhibitor [16,17]. Second-generation drugs such as nilotinib [18,19] and dasatinib [20] (Figure 1A) have been developed that are able to target most, but not all, imatinib-resistance mutations. Currently, third-generation therapeutic agents are in development or clinical evaluation.

A major goal in the further development of kinase inhibitors is to maintain a degree of specificity similar to that of imatinib for Abl, thereby minimizing potential side effects from off-target interactions. Thus, to identify potential secondary targets of these inhibitors, recent studies have focused on chemical proteomics screens for drug-interactors [21,22]. Briefly, the screens involve the generation of matrix-linked inhibitors that are used to pull down interacting proteins, which are then identified by mass spectrometry and validated by binding and activity studies. This approach has been pursued with imatinib and the second-generation inhibitors nilotinib, dasatinib, and bosutinib. In addition to the known targets of these inhibitors, additional kinase targets were identified. For imatinib, the screens also identified a surprising non-kinase target, the oxidoreductase NQO2, which also was shown to be a target of the second-generation inhibitor nilotinib but not dasatinib or bosutinib [21,22].

NQO2 is a cytosolic flavoprotein that carries out the 2-electron reduction of quinones using electron donors such as nicotinamide riboside (NRH). It is one of two closely related cellular quinone reductases (the other being NQO1) and is thought to be involved in metabolic reduction and xenobiotic detoxification [23,24], although its precise physiological role remains uncertain [25]. Interestingly, NQO2 is highly expressed in myeloid cells, the targets of imatinib in CML anticancer therapy, and RNAi knockdown of NQO2 in K562 cells, an immortalized Bcr-Abl-positive CML cell line, resulted in reduced proliferation rates [26]. The phosphorylation of NQO2 on a serine residue in K562 cells was observed [22], suggesting potential regulation of the activity of the enzyme by phosphorylation.

Imatinib and nilotinib inhibited the NQO2-mediated reduction of the anticancer drug mitomycin C, with IC_50 _values of 1 μM for imatinib and 1.8 μM for nilotinib [21]. Another set of experiments demonstrated competitive inhibition by imatinib of the NQO2-mediated reduction of menadione (vitamin K_3_) with a K_i _of 39 nM, in line with an IC_50 _value of 43 nM obtained by competitive binding assay [22]. These data, together with the observation that imatinib levels reach ~1 μM in the serum of patients [14,27], imply that NQO2 inhibition occurs in imatinib-treated CML patients, raising the possibility that NQO2 inhibition may contribute to the overall pharmacological effects of these drugs.

The exact mechanism by which NQO2 is inhibited by imatinib is unknown. Neither chemical proteomics study detected turnover of imatinib by NQO2. One study proposed that imatinib inhibits NQO2 activity via competition with the FAD cofactor for binding to the enzyme [21], while the other study reported competitive inhibition with respect to the substrate menadione [22].

Here, we report studies undertaken to further understand the structural basis for the molecular mechanism by which imatinib binds to and inhibits NQO2. Spectroscopic measurements confirmed the direct binding of imatinib to NQO2, with concurrent changes in the flavin environment indicating that the FAD is not displaced by imatinib. We have solved the X-ray crystal structure of NQO2 bound to imatinib to 1.75 Å resolution, which shows that the drug is bound adjacent to the flavin isoalloxazine ring. The X-ray structure further provides an explanation for the binding specificity of NQO2 for imatinib and nilotinib, as well as for the effects of mutation of the reported phosphorylation sites on NQO2.

## Results and discussion

### Inhibition of NQO2 by imatinib and nilotinib

To assess the inhibition of NQO2 by imatinib, a continuous spectrophotometric assay was used. Briefly, the NQO2-mediated reduction of menadione to menadiol is coupled to the reduction of the dye 3-(4,5-dimethylthiazol-2-yl)-2,5-diphenyltetrazolium bromide (MTT) by menadiol, resulting in an increase in absorbance at 590 nm. Nilotinib, dasatinib, and quercetin, a flavonoid NQO2 inhibitor that is also a non-specific kinase inhibitor [28], were included for comparison. As shown in Figure 1B, imatinib inhibited NQO2 activity with an IC_50 _value of 82 nM, approaching that observed for quercetin (42 nM). These values are in the same range as those determined previously [22,28]. Nilotinib inhibited NQO2 activity with an IC_50 _value of 381 nM, while dasatinib did not inhibit NQO2 activity significantly at the concentrations tested (IC_50 _> 100 μM).

### Binding of imatinib to NQO2

**Figure 2:**
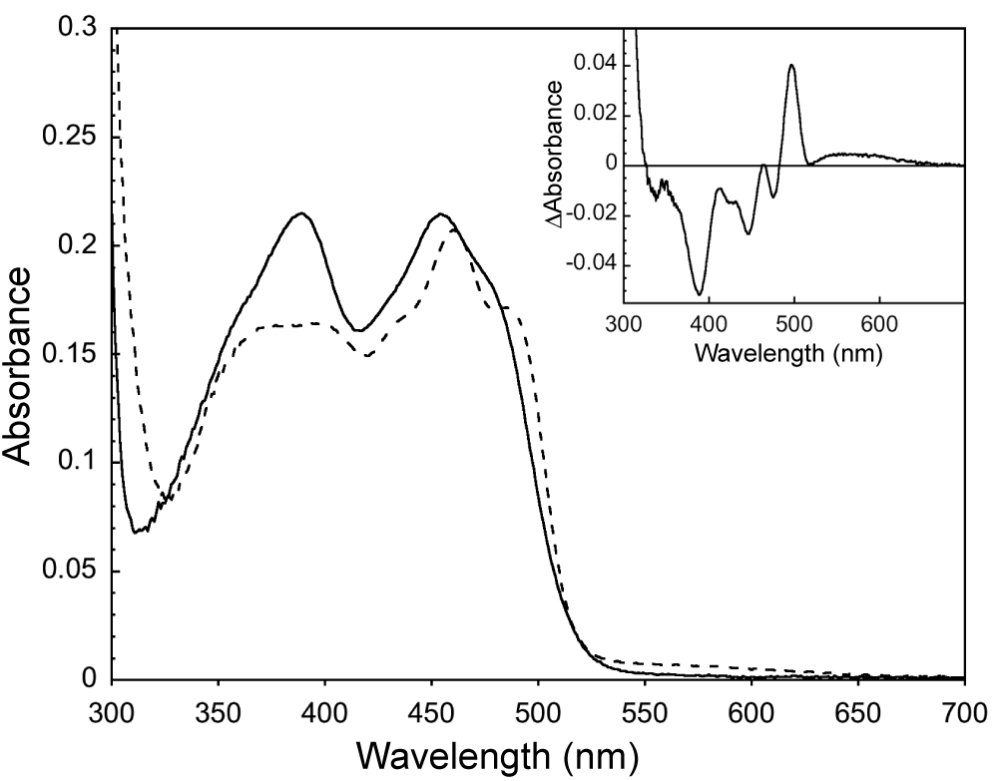
**Binding of imatinib to NQO2**. Binding of imatinib to NQO2 causes significant changes in the electronic absorption spectrum of the flavin cofactor. Shown is the spectrum of 18.3 μM NQO2 in the absence (solid line) and presence (dashed line) of 40 μM imatinib at 25°C. *Inset*, difference spectrum calculated by subtraction of the spectrum of the unbound protein from that of the imatinib-bound protein.

To investigate the mechanism of inhibition of NQO2 by imatinib, we first examined the effect of imatinib binding on the flavin environment using electronic absorption spectroscopy (Figure 2). The absorbance spectrum of the enzyme in the absence of imatinib (Figure 2, solid line) is characteristic of an oxidized flavoprotein [29], with absorption bands at 454 and 389 nm and a shoulder at 477 nm. Upon addition of imatinib, the absorption bands are perturbed significantly (Figure 2, dashed line) and the difference spectrum exhibits minima at 389, 446, and 475 nm and maxima at 496 and 560 nm (Figure 2, inset). Thus, the flavin is affected appreciably by imatinib binding, but not displaced from the protein, as the observed spectrum in the presence of imatinib is not characteristic of free flavin [30]. The results of our spectroscopic investigations are consistent with the notion that imatinib competes with substrate for binding in the active site adjacent to the flavin, as suggested previously [22].

### Structure of the NQO2-imatinib complex

To determine how imatinib binds to NQO2, we crystallized human NQO2 in the presence of imatinib and solved the structure of the complex by molecular replacement using the structure of the ligand-free enzyme (1QR2 [31]). The complex crystallized in space group I422, with one monomer in the asymmetric unit. The crystals diffracted x-rays to a resolution of 1.75 Å. Four N-terminal residues (including two from a purification tag) and one C-terminal residue are disordered. The final model includes residues 3–230 of human NQO2, one FAD molecule, one imatinib molecule, one zinc atom, two (4R)-2-methylpentane-2,4-diol (MRD) molecules, and 213 solvent molecules. We use the same residue numbering scheme for the model reported here that is used for all previous structures of NQO2, in which the genetically encoded N-terminal methionine is excluded, so that the second genetically encoded residue, Ala 2, is labeled as residue 1 in the structure. The refined model has working and free R-values [32] of 15.0% and 18.2%, respectively (Table 1). 97.35% of observed backbone dihedral angles lie in the favored Ramachandran regions, and the remaining 2.65% lie in allowed Ramachandran regions.

**Table 1:** Crystallographic data and refinement statistics

**Data collection**	
Beamline	ALS 8.2.1
Wavelength (Å)	1.2547
Space group	I422
Unit cell	*a *= 100.3 Å, *b *= 100.3 Å, *c *= 104.9 Å α = 90°, β = 90°, γ = 90°
Resolution (Å)*	36.25–1.75 (1.84–1.75)
R_merge _(%)*	8.3 (55.9)
I/σ (I) *	22 (3.9)
Completeness (%)*	99.9 (99.9)
Redundancy*	9.2 (9.3)
**Refinement**	
Unique reflections	27265
Free R test set (% of total data)	5.12
R_work_/R_free _(%)	15.0/18.2
Monomers per asymmetric unit	1
Number of non-hydrogen atoms	2166
Protein	1847
Ligand (imatinib, FAD, MRD)	106
Water	213
r.m.s. deviation, bond lengths (Å)	0.012
r.m.s. deviation, bond angles (Å)	1.093

* Outer resolution shell and statistics for that shell are in parentheses

**Figure 3:**
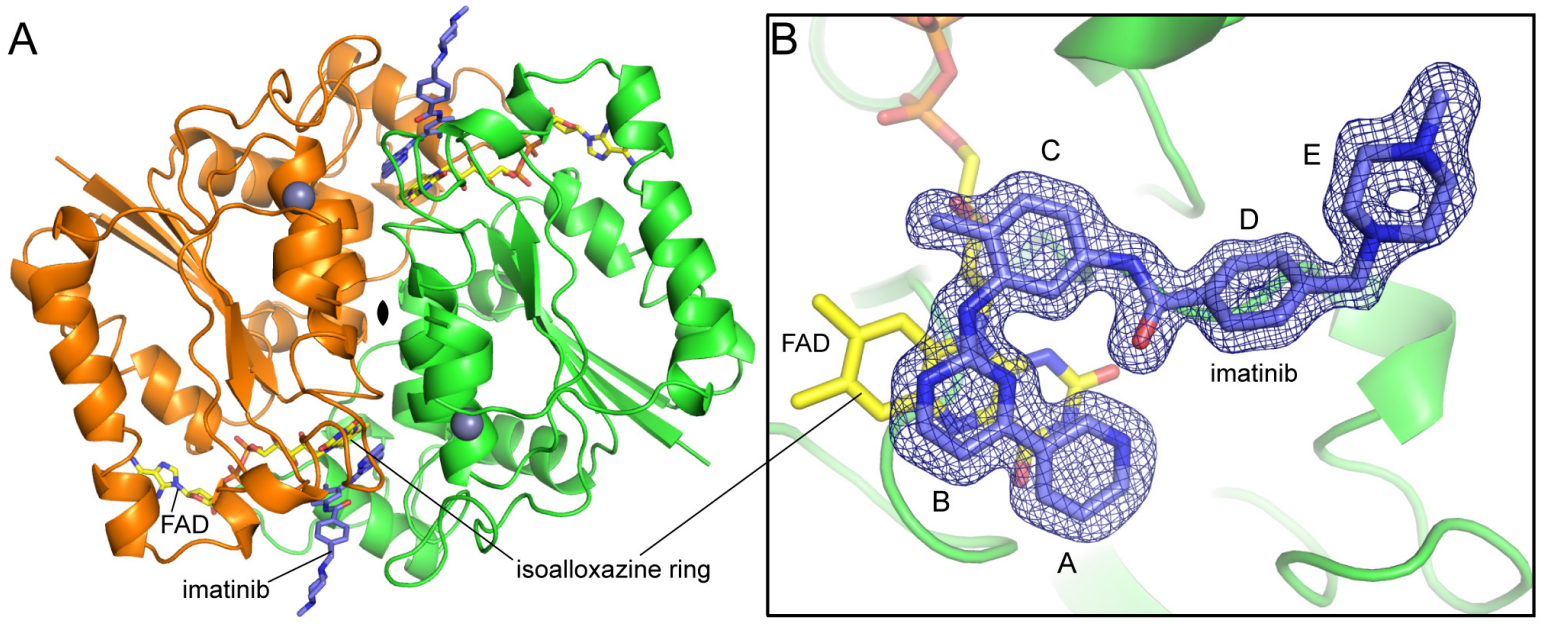
**Structure of NQO2 in complex with imatinib**. A) Cartoon representation of the NQO2 dimer bound to imatinib. The two monomers (colored green and orange) are related by a crystallographic two-fold axis of rotation. B) Difference electron density (contoured at 3.0 σ) from a map calculated with the imatinib ligand removed is shown over the refined model of the imatinib-bound NQO2. In each panel, the FAD and imatinib molecules are depicted as yellow and blue stick figures, respectively; carbon is colored yellow (FAD) or light blue (imatinib); nitrogen, blue; oxygen, red; phosphorus, orange. Bound zinc ions are shown as grey spheres.

**Figure 4:**
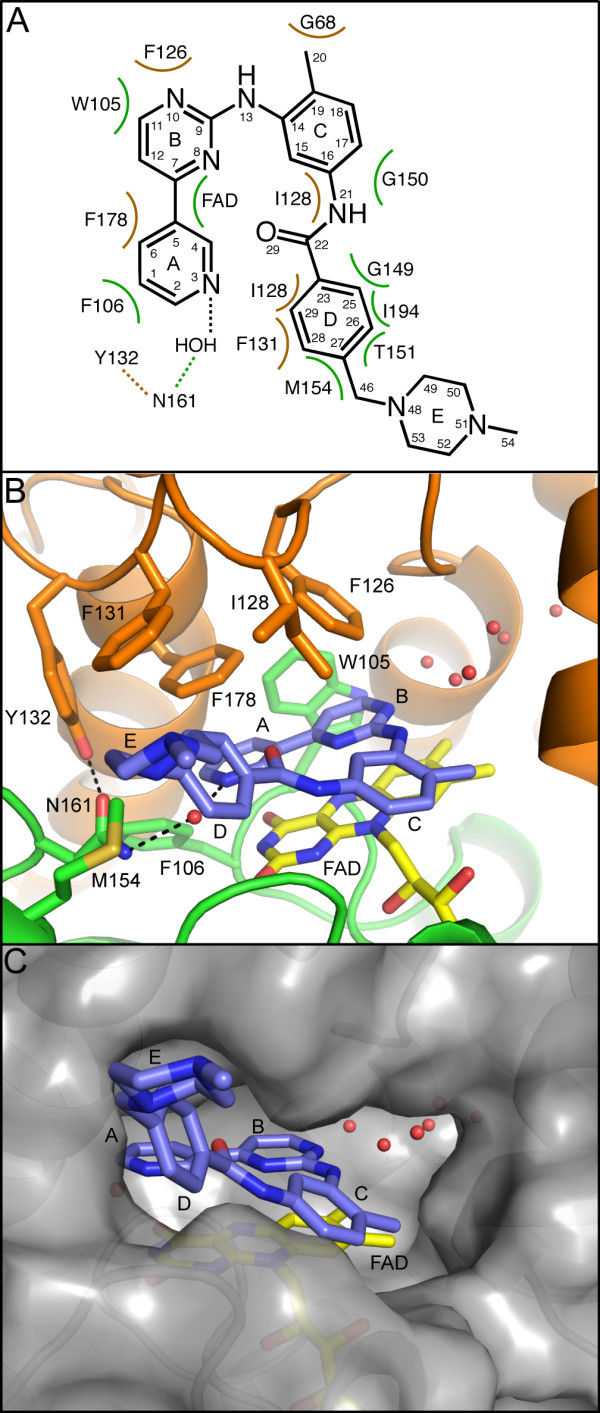
**Interactions of imatinib with NQO2**. A) Schematic of imatinib-NQO2 interactions. Van der Waals interactions of imatinib with NQO2 residues are indicated as semi-circles, and hydrogen bonds are represented by dotted lines. Contacts from each NQO2 monomer are colored green or orange. B) Residues involved in contacts between imatinib and NQO2 are primarily hydrophobic interactions. One water-mediated hydrogen bond is formed between imatinib and the side chain of Asn 161, which is positioned by a hydrogen-bonding interaction with Tyr 132. Residues involved in contacts between imatinib and NQO2 are shown as stick figures, and water molecules are shown as red spheres. Not shown: Gly 149, Gly 150, Thr 151, and Ile 194. C) Surface representation of the active site of NQO2 in complex with the kinase inhibitor imatinib. In each panel, the FAD and imatinib molecules are depicted as yellow and blue stick figures, respectively; nitrogen is colored blue and oxygen, red. Water molecules are shown as red spheres. The imatinib rings are lettered as in Figure 1A.

Biochemical and structural studies have demonstrated that NQO2 is a dimer [31,33]. In our structure, the dimer is formed from two adjacent molecules related by a crystallographic 2-fold axis. Dimerization leads to formation of two FAD-containing active sites per dimer, with each active site located in a deep pocket at the interface between monomers (Figure 3A). Clear density into which imatinib could easily be built was observed in the electron density maps at the active site after molecular replacement (Figure 3B). Imatinib interacts with NQO2 primarily via hydrophobic interactions, as shown in Figure 4A. The isoalloxazine ring of the flavin cofactor forms the floor of the active site, upon which the pyridine and pyrimidine rings (rings A and B) of the bound imatinib stack, with an average distance of ~3.3 Å between atoms in closest contact. The side chains of Trp 105 and Phe 106 form the back of the active site, while three hydrophobic amino acids from the other monomer in the NQO2 dimer, Phe 126, Ile 128, and Phe 178, form the top of the active site (Fig 4B).

Steric constraints prevent the bound imatinib from binding in an extended conformation, causing it to instead adopt a horseshoe shape that directs the methylbenzene, benzamide, and N-methylpiperazine rings (rings C, D and E) away from the active site and towards the solvent (Figure 4C). The imatinib N-methylpiperazine ring extends into solvent and forms crystal contacts with a third symmetry-related molecule.

The inhibitor makes no direct hydrogen bonds with the protein, although several water-mediated hydrogen bonds are observed. A water-mediated hydrogen bond is formed between Asn 161, which is positioned by a hydrogen-bonding interaction with Tyr 132, and the N3 nitrogen of the imatinib pyridine ring (ring A). The N8 and N13 nitrogen atoms of the imatinib 2-aminopyrimidine moiety (ring B) interact with a cluster of ordered waters (Figure 4B). A similar water cluster is also observed in several other NQO2-ligand complexes [26,34], and such water-mediated interactions have been proposed to be important for NQO2 ligand recognition [31,35].

**Figure 5:**
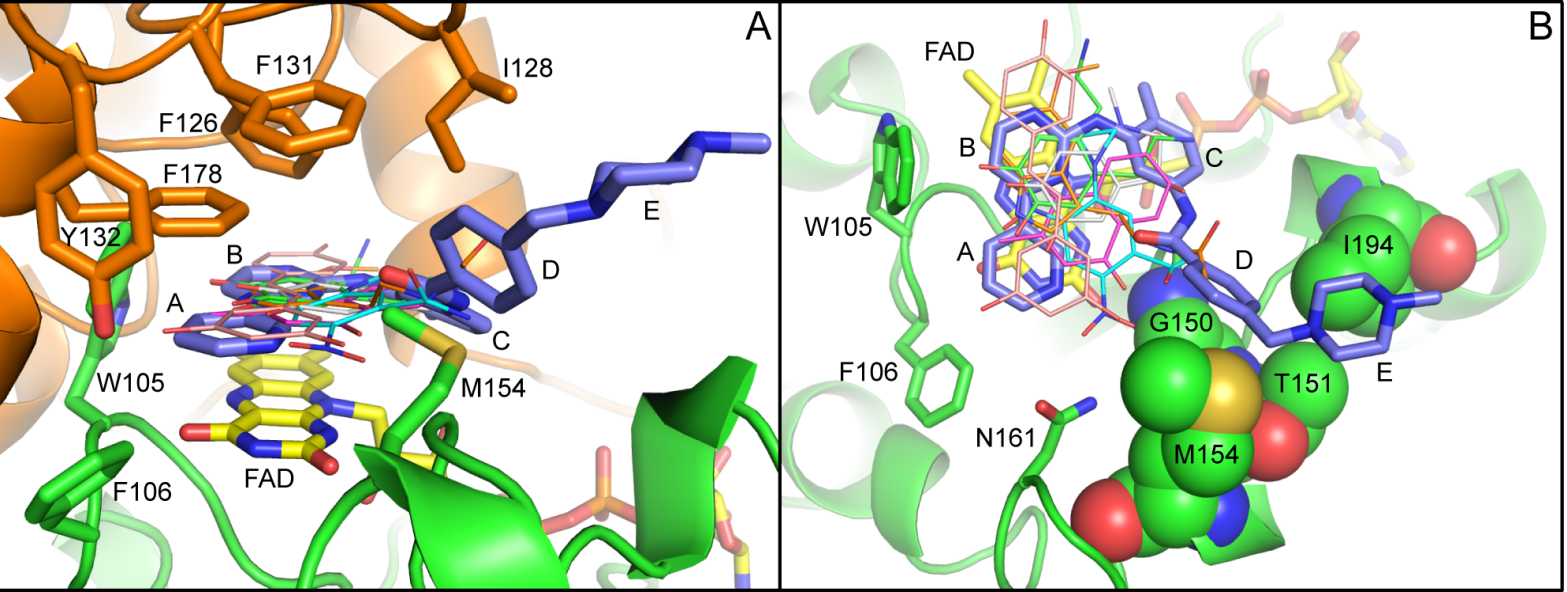
**Comparison between binding of imatinib and other small molecules to NQO2**. A) Overlay of the structures of several substrate- and inhibitor-NQO2 complexes with the imatinib-NQO2 complex. The loop containing Asn 161 has been removed for clarity. All of the bound molecules contain aromatic rings that stack above the flavin isoalloxazine group. B) The same overlay as in A), rotated to show interactions of the imatinib methylbenzene, benzamide, and N-methylpiperazine rings with hydrophobic residues (shown as CPK models) around the rim of the NQO2 active site. In each panel, imatinib (blue), the FAD cofactor (yellow), and several residues important for inhibitor binding are shown as stick figures, while the other overlaid NQO2-bound molecules are shown as line figures. The imatinib rings are lettered as in Figure 1A. The other molecules are menadione (magenta), resveratrol (pink), adrenochrome (grey), dopamine (green), melatonin (orange), and CB1954 (teal), from PDB ID 2QR2[31], 1SG0[26], 2QMY[37], 2QMZ[37], 2QWX[35], and 1XI2[36], respectively.

Substrates and inhibitors of NQO2 contain planar aromatic moieties that insert into the active site and stack on the isoalloxazine ring of the flavin cofactor [26,31,34-37] (Figure 5A). For imatinib this role is played by the 4-pyridyl-2-aminopyrimidine moiety (rings A and B). Because it is substantially larger than previously characterized NQO2 ligands, imatinib forms additional interactions, including hydrophobic interactions between the methylbenzene, benzamide, and N-methylpiperazine rings (rings C, D and E) and several amino acids surrounding the rim of the active site (Figure 5B).

### Discrimination by NQO2 between imatinib, nilotinib and dasatinib

The imatinib binding mode we observe in our structure explains why NQO2 is inhibited by nilotinib, but not by dasatinib [21]. Nilotinib contains a 4-pyridyl-2-phenylaminopyrimidine core (nilotinib rings A, B and C, Figure 1A), identical to that of imatinib, that can adopt a planar conformation and fit in the active site, and a similar amide-linked substituted phenyl ring (nilotinib ring D, Figure 1A), which likely also extends towards solvent from the active site. The modest reduction in affinity relative to imatinib may be due to the increased bulk and decreased flexibility of the nilotinib trifluoromethylbenzene and methylimidazole rings (nilotinib rings D and E, Figure 1A) compared to the benzamide and N-methylpiperazine rings of imatinib (imatinib rings D and E, Figure 1A). The chemical structure of dasatinib contains an aminopyrimidine core similar to that of imatinib and nilotinib (dasatinib ring B, Figure 1A), but the adjacent non-aromatic hydroxyethylpiperazine ring (dasatinib ring A, Figure 1A) cannot adopt the planar conformation necessary for stacking onto the flavin isoalloxazine ring. Dasatinib is unable to adopt a *cis *conformation (see the kinase discussion below for an explanation) around the bond between the aminopyrimidine and thiazole rings (dasatinib rings B and C, Figure 1A) that is capable of productive interaction with the rim of the active site.

### Specificity of imatinib for NQO2 over NQO1

**Figure 6:**
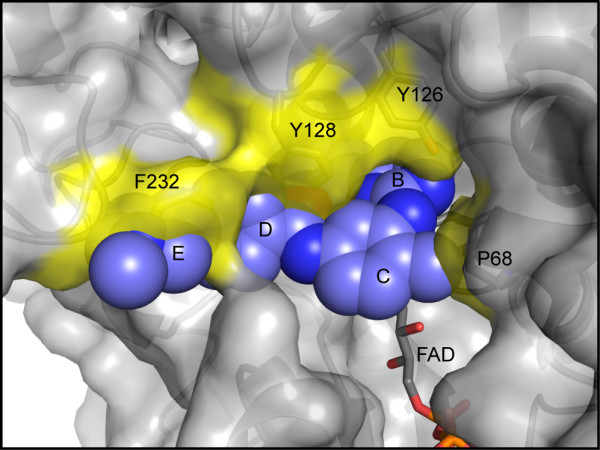
**NQO1 active site is incompatible with imatinib binding**. A) Model of imatinib in the active site of NQO1. The model was generated by superimposing the structure of human NQO1 (PDB ID 1D4A) onto the structure of the NQO2-imatinib complex. Imatinib (blue) is shown as a CPK model, while NQO1 is shown in surface and cartoon representations, with the FAD cofactor and selected residues depicted as stick figures. The imatinib rings are lettered as in Figure 1A. Potential clashes between NQO1 residues and imatinib are highlighted in yellow.

NQO2 is closely related to another quinone reductase, NQO1. Despite catalyzing the same reaction, namely, the two-electron reduction of quinones, and sharing 49% identity at the amino acid level [33], NQO1 is not inhibited by imatinib [22]. A comparison of the structures of human NQO1 [38] with the structure of imatinib-bound NQO2 described here provides an explanation for this observation. While the structures of NQO1 and NQO2 superimpose well, with a r. m. s. deviation of 0.770 Å over 220 Cα atoms, NQO2 lacks a C-terminal domain of 43 amino acids. The C-terminal domain of NQO1 is involved in binding of the adenosine and diphosphate moieties of the cosubstrate NAD(P)H, which is not used by NQO2 [28,39]. When the two structures are superimposed, the side chain of Phe 232 in the C-terminal domain of NQO1 occupies the space in which the imatinib N-methylpiperazine ring (ring E, Figure 6) is found in the NQO2 structure. In addition, the side chains of Tyr 128 and Pro 68 at the rim of the NQO1 active site occlude the space that is occupied in the NQO2 structure by the imatinib benzamide and methylbenzenes rings (rings D and C), respectively, and the side chain hydroxyl group of Tyr 126 clashes with the imatinib aminopyrimidine ring (ring B). Thus, steric hindrance by residues in the C-terminal domain unique to NQO1, and by residues in the active site that differ between NQO1 and NQO2, prevents imatinib binding in the NQO1 active site.

### Comparison of the imatinib binding modes observed in NQO2 and in kinases

**Figure 7:**
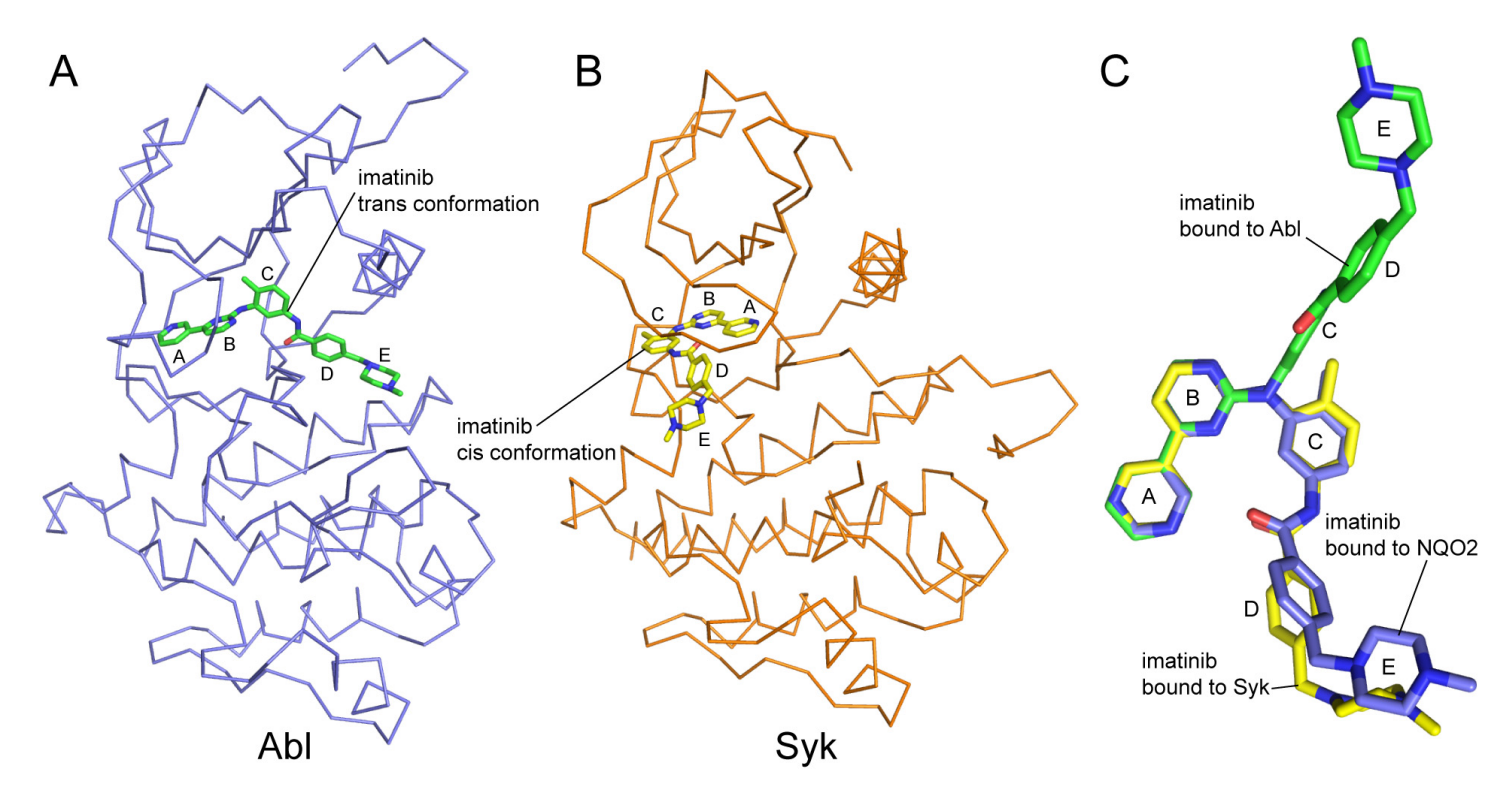
**Comparison between imatinib-NQO2 and imatinib-kinase binding modes**. A) The structure of imatinib bound to the kinase domain of Abl (PDB ID 1IEP) [6]. Imatinib binds in an extended conformation, with the pyridylpyrimidine moiety (rings A and B) *trans *to the methylbenzene and benzamide rings (rings C and D). B) The structure of imatinib bound to the kinase domain of Syk (PDB ID 1XBB) [44]. Imatinib binds in a compact conformation, with the pyridylpyrimidine moiety *cis *to the methylbenzene and benzamide rings. C) The conformation of imatinib bound to NQO2 most resembles the *cis *kinase-binding conformation. Shown are the structures of imatinib from the Syk complex 1XBB (yellow), the Abl complex (green), and the NQO2 complex (blue), with their pyridylpyrimidine moieties superimposed. In all panels, proteins are shown in ribbon representation and imatinib is shown as a stick model. The imatinib rings are lettered as in Figure 1A.

In the structures of imatinib bound to its primary pharmacological target Abl [6-8,40], as well as to several other kinases [10,41,42], the inhibitor binds in an extended conformation, with the pyridylpyrimidine moiety (rings A and B) *trans *to the methylbenzene and benzamide rings (rings C and D) with respect to the C9-N13 bond (Fig 7A). Nilotinib also binds to Abl in a similar extended conformation [43]. Imatinib can also bind in a more compact conformation, with the pyridylpyrimidine moiety *cis *to the methylbenzene and benzamide rings (Fig 7B), as seen in the structure of imatinib bound to Syk [44] and in the structure of a desmethyl imatinib analogue bound to Src [45]. This folded-over conformation is less common, and is likely to correspond to a low-affinity interaction because imatinib has limited efficacy against Syk [44]. The conformation of the imatinib molecule in complex with NQO2 resembles this *cis *conformation (Fig 7C). This may have implications for the design of other pyridylpyrimidine-containing kinase inhibitors and other drugs, which might display unintended interaction with NQO2 if they depend on or are capable of adopting a similar *cis*-like conformation.

### NQO2 phosphorylation

**Figure 8:**
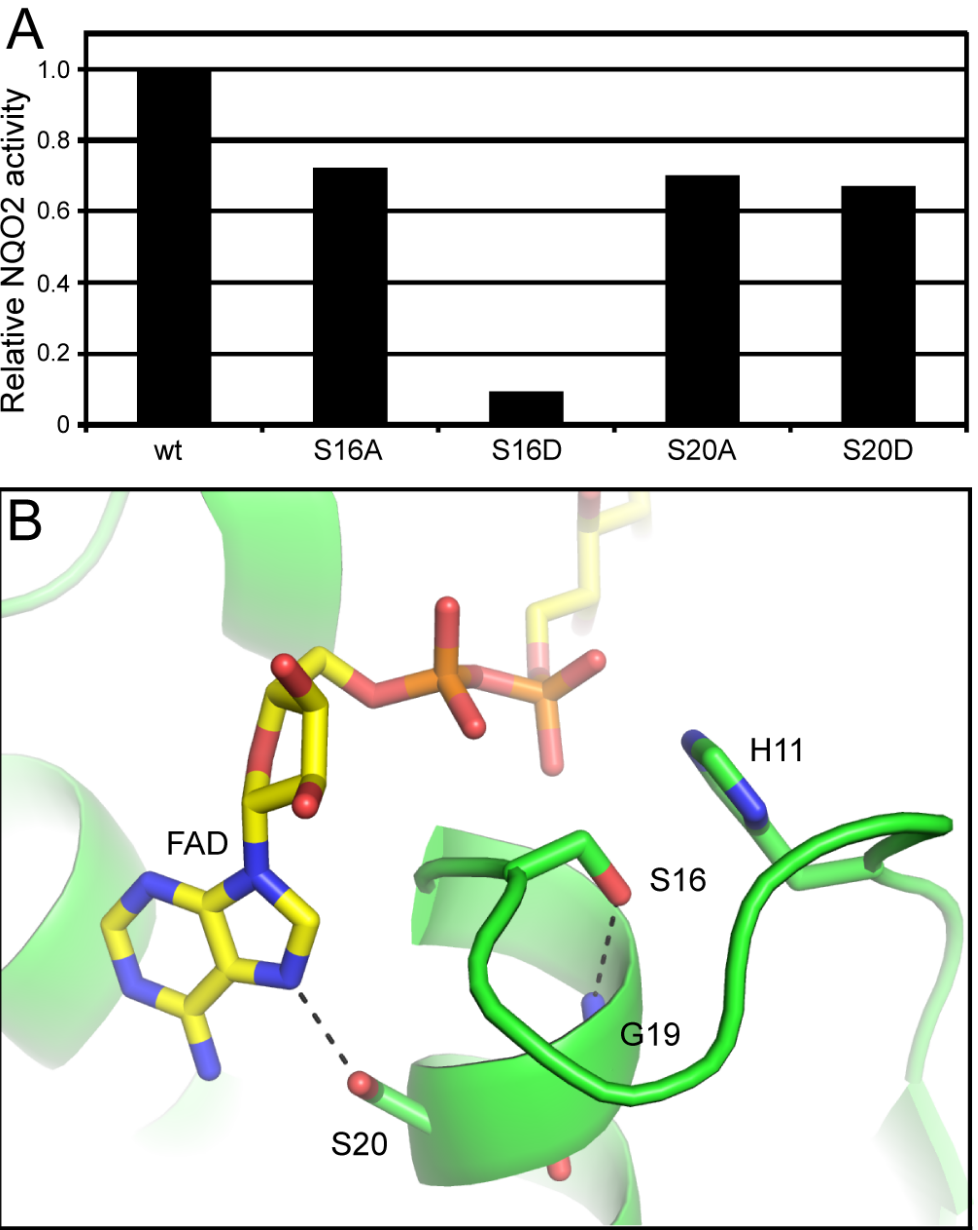
**Analysis of potential NQO2 phosphorylation sites**. A) Relative NQO2 activities of putative phosphorylation site mutants. Mutation of either Ser 16 or Ser 20 results in diminished activity, with the phosphorylation-mimicking S16D mutation having the most drastic effect. B) Ser 16 and Ser 20 are located next to the binding site for adenine and diphosphate moieties of the FAD cofactor. Ser 20 is solvent-exposed and involved in recognition of the FAD adenine moiety, while Ser 16 is mostly buried and involved in interactions that help form the adenine binding site. The FAD cofactor (yellow) and selected residues (green) are shown as stick models. Hydrogen bonds are depicted as dashed lines.

NQO2 is phosphorylated on either Ser 16 or Ser 20 in the Bcr-Abl-positive cell line K562 [22]. To examine the potential role of this modification in regulation of NQO2 activity, we mutated each residue to Ala or to phosphoserine-mimicking Asp, purified the resulting proteins, and measured their activities. As shown in Figure 8A, the S16A, S20A, and S20D mutants exhibited ~70% of the activity of the wild-type enzyme, while the activity of the S16D mutant was reduced to ~10% of wild-type enzyme activity. Additionally, the S16D mutant was colorless as purified, as opposed to the yellow color displayed by the other mutants and the wild-type protein, and was found to be a mixture of monomer and dimer by analytical gel filtration (data not shown).

Ser 16 and Ser 20 are both located adjacent to the binding site for the FAD cofactor. Ser 20 is involved in recognition of the FAD adenine ring (Figure 8B), so mutation at this position might disrupt this interaction and reduce FAD binding affinity, resulting in the lower activities of the S20A and S20D mutants (Figure 8A). The side chain of Ser 16 forms a hydrogen bond with the main chain amide of Gly 19 and packs against the imidazole side chain of His 11, keeping the main chain of Ser 16 from blocking part of the FAD adenine binding site. The Ser 16 hydroxyl group is also close to the diphosphate moiety of the FAD (3.2 – 3.6 Å from the phosphate oxygen atoms to the Ser 16 Cβ atom) (Figure 8B). The S16A mutation might disrupt the hydrogen-bonding and van der Waals interactions necessary for proper formation of the adenine binding pocket, resulting in a decrease in FAD binding affinity similar to that caused by the Ser 20 mutations. The pronounced drop in activity of the S16D mutant (Figure 8A), as well as the observed loss of yellow color and partial monomerization, suggests loss of the FAD cofactor. Such cofactor loss might result from repulsion between the phosphate oxygen atoms of the FAD diphosphate moiety and the aspartate side chain of the S16D mutant, in addition to disruption of interactions necessary for proper formation of the adenine binding pocket.

What role could phosphorylation of NQO2 play *in vivo*? While the large decrease in the activity of the S16D mutant suggests that phosphorylation of this residue could mediate down-regulation of NQO2 activity, the buried nature of the Ser 16 side chain likely precludes it from being a target for phosphorylation in cells. Furthermore, studies have indicated that NQO2 is functional in K562 cells [26] – the same cells in which NQO2 was found to be phosphorylated [22], suggesting that phosphorylation of NQO2 is unlikely to be directly inactivating. Thus, it is more likely that the solvent-exposed Ser 20 is the site of NQO2 phosphoryation. The relatively minor effect of Ser 20 mutation on enzyme activity suggests that phosphorylation may regulate some other aspect of NQO2 function, rather than by directly affecting enzyme activity. It is worth noting that imatinib, and therefore Bcr-Abl inhibition, did not alter the phosphorylation of NQO2 on Ser 20 [22]. Therefore, the kinase that phosphorylates NQO2 is unlikely to be a direct Bcr-Abl effector.

## Conclusion

To date, numerous crystal structures of the flavoprotein oxidoreductase NQO2 in complex with quinones, natural products, and xenobiotics have been solved [26,31,34-37], and we report here the x-ray crystal structure of NQO2 bound to the leukemia drug imatinib, an inhibitor of Bcr-Abl. Our study has been motivated by the possibility that inhibitors of Bcr-Abl might also interact with unintended targets, and that these interactions may lead to side effects during drug treatment. The data reported in this work elucidate the mechanism of NQO2 inhibition by imatinib, and suggest that NQO2 is likely to be inhibited by imatinib at the 1-μM serum concentration typical in patients [14,27]. The question remains as to what the physiological consequences of NQO2 inhibition by imatinib may be and whether they contribute to the efficacy of imatinib in treatment of CML. While it has been reported that RNAi knockdown of NQO2 or treatment with the polyphenol NQO2 inhibitor resveratrol results in a reduction of proliferation of Bcr-Abl-positive K562 cells [26], we failed to see any effect of NQO2 knockdown on K562 cell proliferation (data not shown). On the other hand, NQO2 knockout mice exhibit myeloid hyperplasia [46] and increased susceptibility to chemical carcinogenesis [47]. Although these side effects have not been observed yet in CML patients treated with imatinib [48,49], future studies on the effects of NQO2 inhibition on cellular function and the role of NQO2 in cancer initiation and progression may shed light on the possible physiological consequences of NQO2 inhibition by imatinib or nilotinib.

## Methods

### Expression and purification of recombinant NQO2

Full-length human NQO2 was cloned into the vector pETM30 (EMBL-Heidelberg, Protein Expression Facility), resulting in a His6-GST-NQO2 fusion protein with a TEV protease cleavage site between GST and NQO2. The construct was verified by sequencing and transformed into Tuner(DE3)pLysS cells (Novagen) for protein expression. Cells from 2 × 1 L expression cultures were harvested by centrifugation, and cells were resuspended in buffer A (50 mM Tris-HCl pH 8.0, 300 mM NaCl, 10 mM imidazole). Cells were lysed by sonication, and the lysate was cleared by centrifugation at 200,000 g. The supernatant was applied to a 5 mL HisTrap FF column (GE Healthcare) equilibrated with buffer A, washed with buffer A until the absorbance at 280 nm was stable, and eluted with 250 mM imidazole in buffer A. The eluted yellow protein was desalted into buffer A and the His6-GST purification tag was cleaved off using TEV protease. The protease and His6-GST were removed from the NQO2 by passage back over the HisTrap column. NQO2 was desalted into buffer B (25 mM Tris-HCl pH 8.0, 25 mM NaCl) by concentration/dilution on a 5 K Amicon Ultrafree concentrator (Millipore) and loaded onto a 5 mL HighTrap Q HP column (GE Healthcare). Protein was eluted with a gradient of 0 – 500 mM NaCl in buffer B, and fractions containing NQO2 (as ascertained by SDS-PAGE) were pooled, concentrated on a 5 K Amicon Ultrafree concentrator, and stored at -20°C.

### NQO2 activity assays

Activity of NQO2 was assayed using a continuous spectrophotometric assay, adapted from previously reported methods [22,28], with menadione as substrate and 1-carbamoylmethyl-3-carbamoyl-1,4-dihydropyrimidine (CCHP) as co-substrate. Reduction of the dye 3-(4,5-dimethylthiazol-2-yl)-2,5-diphenyltetrazolium-bromide (MTT) was monitored at 590 nm using a SpectraMax M5 plate reader (Molecular Dynamics). Reaction were carried out at 30°C in 200 μl containing 25 mM Tris-HCl pH 7.5, 0.01% Tween-20, 0.18 mg/ml BSA, 1 μM FAD, 100 μM menadione, 0.134 mg/ml MTT, and 500 μM CCHP. Reactions were initiated by addition of 20 ng recombinant NQO2. For NQO2 inhibition assays, the respective inhibitors were pre-incubated with the recombinant NQO2 and reactions were started by the addition of substrate/co-substrate. Slopes of the absorption-time-diagram were used to calculate the specific activity. Each experiment was repeated twice.

### Electronic absorption spectroscopy

Imatinib stocks were prepared in dimethyl sulfoxide (DMSO). NQO2 (18.3 μM) in 50 mM Tris-HCl pH 7.5 was mixed with imatinib (40 μM in 5% DMSO) or DMSO and placed in a quartz cuvette. Electronic absorption spectra were collected with a Cary 3E spectrophotometer (Varian) at 25°C. Data were collected over the range of 260–700 nm at 600 nm/min with a 1 nm data point interval. A buffer baseline was subtracted for each spectrum. Imatinib exhibits some absorption in this wavelength range (data not shown), so the buffer for the imatinib sample baseline also contained 40 μM imatinib. Difference spectra were obtained by subtraction of the spectrum for the sample containing NQO2 alone from the spectrum of the sample containing imatinib.

### Crystallization and X-ray data collection

NQO2 was thawed and desalted into 50 mM Tris-HCl pH 8.0, by three cycles of concentration/dilution on a 5 K Amicon Ultrafree concentrator. Imatinib stocks were prepared in DMSO. The NQO2-imatinib complex was formed in a solution containing 50 mM Tris-HCl pH 8.0, 5 mM tris(2-carboxyethyl)phosphine, 1.15 mM NQO2, and 1.5 mM imatinib, with a final concentration of 5% DMSO. Crystals of the NQO2-imatinib complex were grown in hanging drops at 20°C using the vapor diffusion method. A volume of 1 μL of protein solution was mixed with an equal volume of reservoir solution [62% (4R, S)-2-methylpentane-2,4-diol (MPD) in 100 mM MES pH 6.0–6.2, 15 μM FAD] and allowed to equilibrate, and yellow rod-shaped crystals grew overnight. For X-ray diffraction experiments, crystals were transferred to a solution of mother liquor (which already contained the cryoprotectant MPD at a concentration of 62%), frozen, and stored in liquid nitrogen. Diffraction data were collected at the Advanced Light Source (Lawrence Berkeley National Laboratory) beamline 8.2.1. Reflections were processed in space group I422 with MOSFLM [50] and SCALA [51]. The structure was determined with the molecular replacement program PHASER [52] using human NQO2 (PDB ID 1QR2) [31] as the search model. The structure was built using ARP/wARP [53], the model was improved using COOT [54], and refinement was carried out using PHENIX [55]. Model quality was analyzed using MOLPROBITY [56], and figures were drawn using the software PYMOL [57]. The atomic coordinates and structure factors have been deposited in the Protein Data Bank (3FW1).

## Authors' contributions

JAW designed and performed research, analyzed data, and drafted the manuscript. OH designed and performed research, analyzed data, and drafted the manuscript. GS-F designed research and analyzed data. JK designed research, analyzed data, and drafted the manuscript. All authors read and approved the final manuscript.
